# Supplementary material for: Distinct Minor Splicing Patterns across Cancers
Source: Genes (Basel). 2022 Feb 21;13(2):387. doi: 10.3390/genes13020387 (PMC8871696; doi:10.3390/genes13020387)
Supplement: Supplementary file 1 [file genes-13-00387-s001.zip › genes-1551931-Supplementary Figures.pdf]

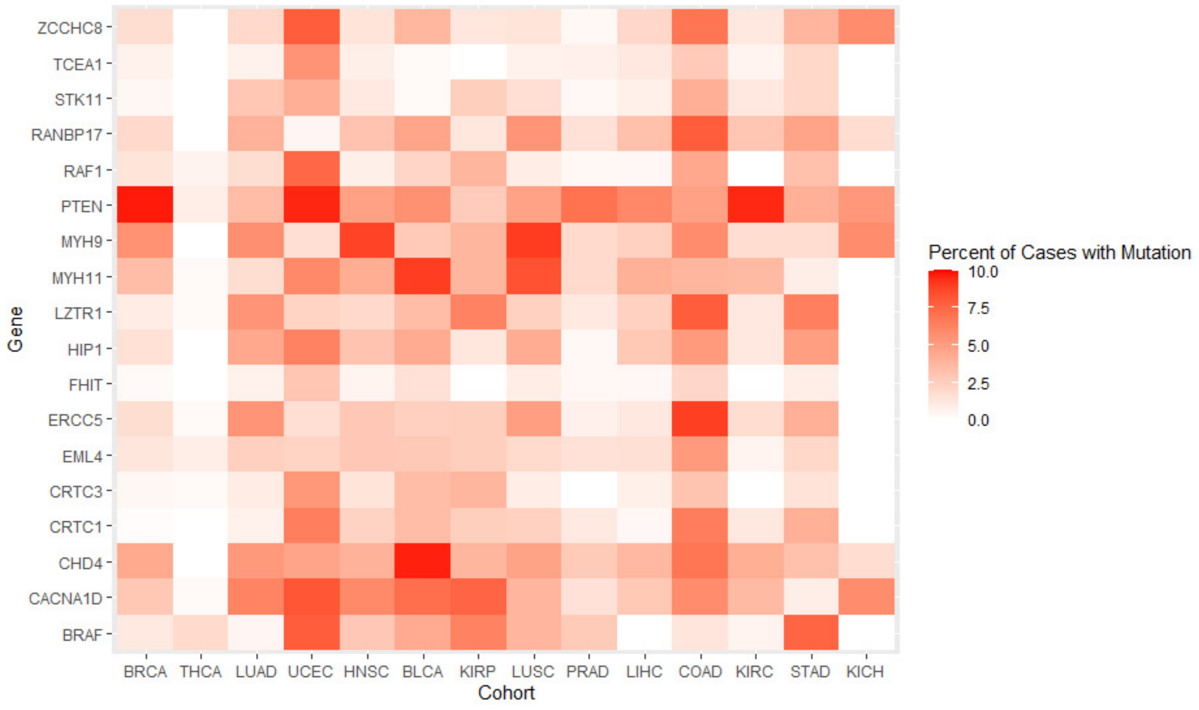

Supplementary Figure S1. Percent of cases with mutated MIGs across TCGA cohorts. Heatmap shows percent of cases with mutated MIG within the given cohort. The relationship is demonstrated by color; white to red where white denotes a low percent of cases while red denotes significant a percent of cases. Cohorts are ordered by their minor splicing score.

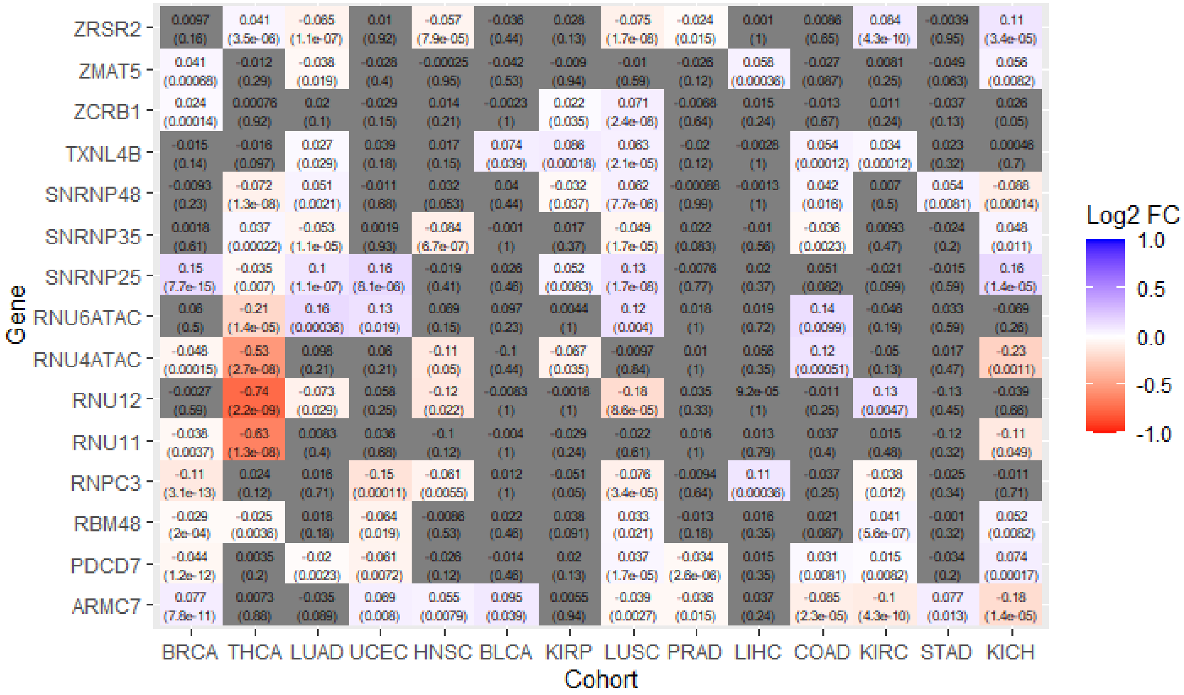

Supplementary Figure S2. Differential expression of minor spliceosome components across TCGA cohorts. Heatmap shows log2 fold change (tumor/normal) in component expression within the given cohort; associated FDR in parentheses. The relationship is demonstrated by color; blue to red where blue denotes significant upregulation in tumor while red denotes significant downregulation in tumor, and gray denotes no significant changes. Cohorts are ordered by their minor splicing score.

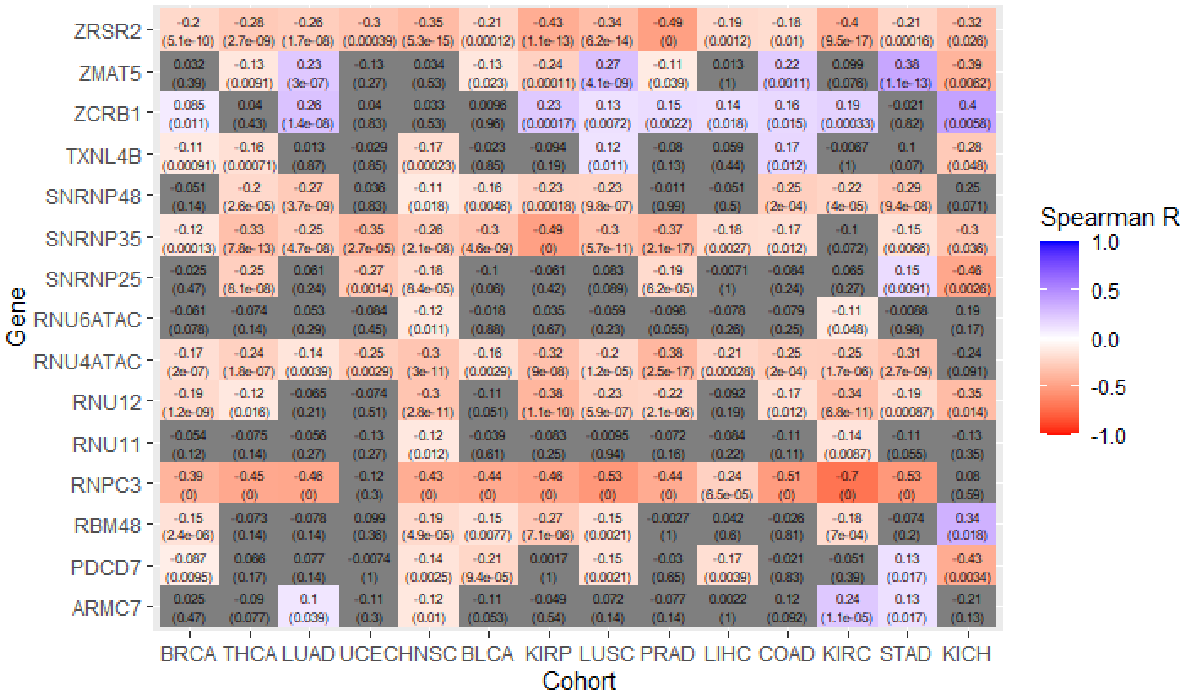

Supplementary Figure S3. Correlation of minor splicing activity in tumors with expression of minor spliceosome components across TCGA cohorts. Heatmap shows Spearman correlation of minor splicing activity with component expression in tumors within the given cohort; associated FDR in parentheses. The relationship is demonstrated by color; blue to red where blue denotes a significant positive correlation while red denotes a significant negative correlation, and gray denotes a non-significant correlation. Cohorts are ordered by their minor splicing score.

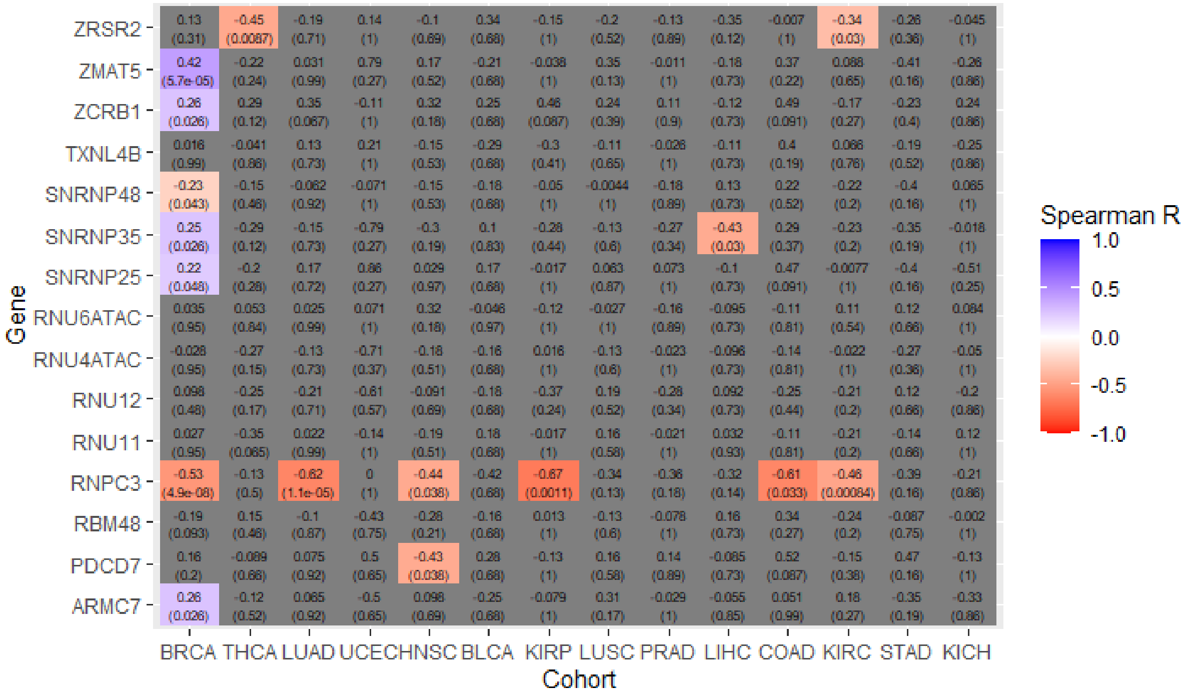

Supplementary Figure S4. Correlation of changes in minor splicing activity with changes in expression of minor spliceosome components between matched samples across TCGA cohorts. Heatmap shows Spearman correlation of changes in minor splicing activity with changes in component expression between matched samples within the given cohort, associated FDR in parentheses. The relationship is demonstrated by color; blue to red where blue denotes a significant positive correlation while red denotes a significant negative correlation, and gray denotes a non-significant correlation. Cohorts are ordered by their minor splicing score.

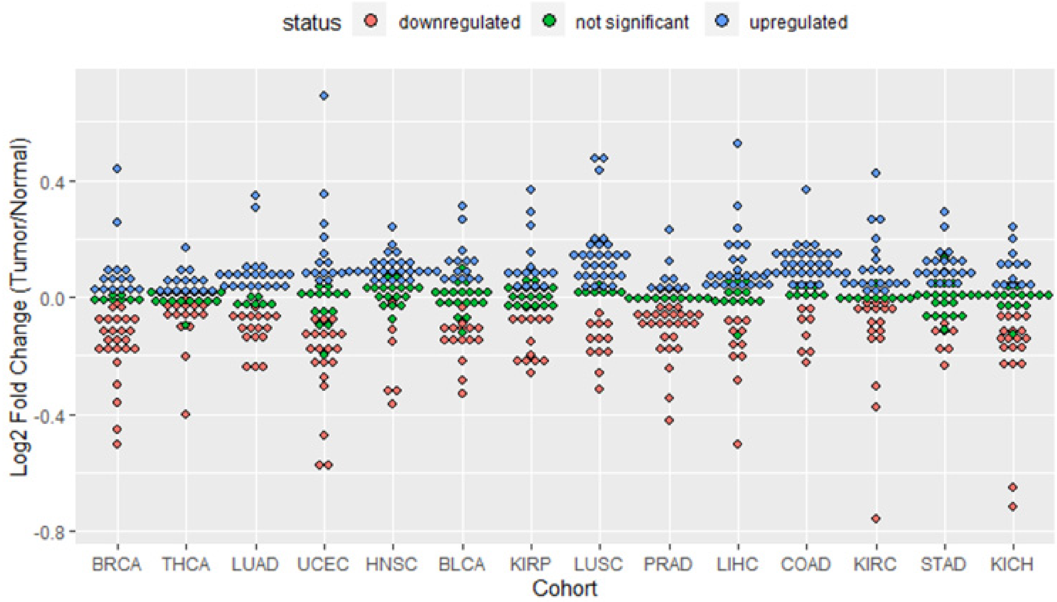

# of Patients	110	58	57	7	42	19	31	49	51	50	24	72	27	23
# of Significant Genes	40	30	39	34	31	29	29	41	35	35	40	38	27	32
# of Upregulated Genes	15	15	22	14	25	14	15	29	9	24	32	21	20	13
# of Downregulated Genes	25	15	17	20	6	15	14	12	26	11	8	17	7	19
Stem Score	-10	0	5	-6	19	-1	1	17	-17	13	24	4	13	-6

Supplementary Figure S5. Expression fold change between tumor and adjacent normal tissue of stem cell genes. Each dot represents the average log2 fold change of a gene(tumor/normal) within the given cohort. Paired Wilcoxon signed-rank test was performed to determine significance, FDR < 0.05.
